# Effect of Oral Peritumoral Tissue on Infiltration and Differentiation of Tumor-Associated Macrophages in Oral Squamous Cell Carcinoma

**DOI:** 10.3390/cells14181481

**Published:** 2025-09-22

**Authors:** Tianyan Piao, Kiyofumi Takabatake, Takuma Arashima, Yulu Zhao, Hotaka Kawai, Htoo Shwe Eain, Yamin Soe, Zin Zin Min, Keisuke Nakano, Hitoshi Nagatsuka

**Affiliations:** Department of Oral Pathology and Medicine, Graduate School of Medicine, Dentistry and Pharmaceutical Sciences, Okayama University, Okayama 700-8525, Japan; pfd12zwy@s.okayama-u.ac.jp (T.P.); de428001@s.okayama-u.ac.jp (T.A.); pm593ijm@s.okayama-u.ac.jp (Y.Z.); de18018@s.okayama-u.ac.jp (H.K.); pmp61kpp@s.okayama-u.ac.jp (H.S.E.); pki31ld5@s.okayama-u.ac.jp (Y.S.); pobk1heq@s.okayama-u.ac.jp (Z.Z.M.); pir19btp@okayama-u.ac.jp (K.N.); jin@okayama-u.ac.jp (H.N.)

**Keywords:** oral squamous cell carcinoma (OSCC), gingival connective tissue cells (GCTCs), periodontal ligament cells (PDLCs), tumor-associated macrophages (TAMs), macrophage polarity, tumor microenvironment (TME)

## Abstract

The recruitment of tumor-associated macrophages (TAMs) in the tumor microenvironment (TME) of oral squamous carcinoma (OSCC) affects significant cancer invasion; however, in the normal host tissue that is located in the cancer’s surrounding area, this is poorly investigated. In this study, we examined the impact of gingival connective tissue cells (GCTCs) and periodontal ligament cells (PDLCs), which are involved in the invasive pathway of OSCC, on oral cancer invasion via TAMs recruitment. Transwell (migration) assays were used to examine the effects of GCTCs and PDLCs on the migration of macrophages, which indicated that the interaction between GCTCs and HSC-2/HSC-3 (human oral squamous cell carcinoma cell line) promoted the recruitment of macrophages, whereas the interaction between PDLCs was inhibited. An indirect co-culture was then used to examine the effects of GCTCs and PDLCs on the differentiation of macrophages, which indicated that the interaction between GCTCs enhanced their ability to transform into M2-type macrophages. Furthermore, the effects of GCTCs and PDLCs on the recruitment of CD45(+) monocytes, F4/80(+) M0 macrophages, iNOS(+) M1 macrophages, and CD163(+) M2 TAMs were assayed by immunohistochemistry. The results revealed that the interaction between GCTCs and HSC-2/HSC-3 promoted the infiltration of CD45(+) monocytes, F4/80(+) M0 macrophages, and CD163(+) M2 TAMs, whereas the PDLCs inhibited it, while their effect on iNOS(+) M1 macrophages was limited. Collectively, the GCTCs contributed to the infiltration of TAMs into the TME of OSCC cells, whereas the PDLCs exerted an inhibitory effect. These findings suggest a potential regulatory mechanism underlying the progression of OSCC.

## 1. Introduction

Solid tumors form a parenchyma–stroma complex, known as the tumor microenvironment (TME). The TME participates in the regulation of tumor progression, invasion, and metastasis [1,2]. Oral squamous cell carcinoma (OSCC), which is the most common malignant neoplasm of the oral cavity [3,4], is a malignant epithelial tumor; however, it is similar to many other solid tumors, wherein infiltration and proliferation affect the interaction between the tumor parenchyma and stroma [5]. Macrophages, together with fibroblasts, vascular endothelial cells, and immune cells, are important cells that form the TME [6]. Macrophages are differentiated into two types: M1 (activating immunity; CD68+, CD80+, and CD86+) with antitumor responses, and M2 (suppressing immunity; CD163+ and CD206+). Tumor-associated macrophages (TAMs) derived from blood monocytes, myeloid-derived suppressor cells, and tissue resident macrophages are induced and differentiated in tumor tissues by cytokines such as C-C motif chemokine ligand 2 (CCL2), CCL5, and colony-stimulating factor 1 (CSF-1) [7]. As the main component of the cancer stroma, TAMs are divided into M1 (classically activated) and M2 (alternatively activated) phenotypes [8]. The M1-type TAMs kill cancer cells by activating immune reactions, whereas the M2-type TAMs contribute to cancer development by suppressing immune reactions [9]. TAMs that enter tumors generally have the characteristics of M2 macrophages and often promote tumor growth and angiogenesis [10,11]; M2 macrophages are believed to contribute to the progression of various types of cancer [8].

The TME of OSCC also contributes to tumor progression through interactions between the cancer parenchyma and cancer stroma; previous studies discuss the association between TAMs and tumor progression, focusing only on the direct interactions between cancer cells and TAMs. TAMs are associated with OSCC progression via the C-X-C motif chemokine ligand 12 (CXCL12)/C-X-C motif chemokine receptor 4 (CXCR4) pathway, high mobility group box 1 (HMGB1), and Epidermal growth factor (EGF) production [12,13,14]. Our previous study indicated that cancer stroma could contribute to the progression of OSCC [15]. During cancer development, oral cancer cells can also encounter normal tissue, such as gingival connective tissue cells (GCTCs) and periodontal ligament cells (PDLCs). Our previous study showed that GCTCs promoted OSCC invasion, whereas PDLCs inhibited poorly differentiated OSCC invasion, which is caused by the different effects of GCTCs and PDLCs on the degree of OSCC differentiation [16,17]. However, the details of the mechanism are unknown.

Therefore, in this study, we hypothesized that the different invasion abilities of OSCC in GCTCs and PDLCs may be due to the different effects on TAMs recruitment and the differentiation of macrophages in the OSCC TME. To confirm this hypothesis, GCTCs and PDLCs were isolated from patients with wisdom teeth, HSC-2 and HSC-3 were selected as the cancer cell model, and RAW264.7 were selected as the macrophage model to assay the effect of GCTCs and PDLCs on the infiltration and differentiation of TAMs in the TME of OSCC. These findings suggested a new potential regulatory mechanism for the progression of OSCC.

## 2. Materials and Methods

### 2.1. Cells and Cell Culture

Human oral squamous carcinoma HSC-2 (JCRB0622) and HSC-3 (JCRB0623), as well as the mouse macrophage cell line RAW264.7 (RCB0535), were procured from the Japanese Collection of Research Bioresources Cell Bank (JCRB) and the RIKEN BioResource Center Cell Bank, respectively. GCTCs and PDLCs were isolated from the normal gingival connective tissue and root surface, respectively, of a 50-year-old male patient and stored as previously described [16]. The HSC-2, HSC-3, and RAW264.7 cells, as well as GCTCs and PDLCs, were maintained in α-MEM (Thermo Fisher Scientific, Inc., Waltham, MA, USA) that was supplemented with 10% FBS and 1% antimycotic–antibiotic (Thermo Fisher Scientific, Inc.) at 37 °C, 5% carbon dioxide, and 95% air in a humidified environment. This study was approved by the Ethics Committee of Okayama University (project identification code:1703-042-001), and written informed consent was obtained from all patients.

### 2.2. Transwell (Migration) Assay and Giemsa Staining

**Figure 1:**
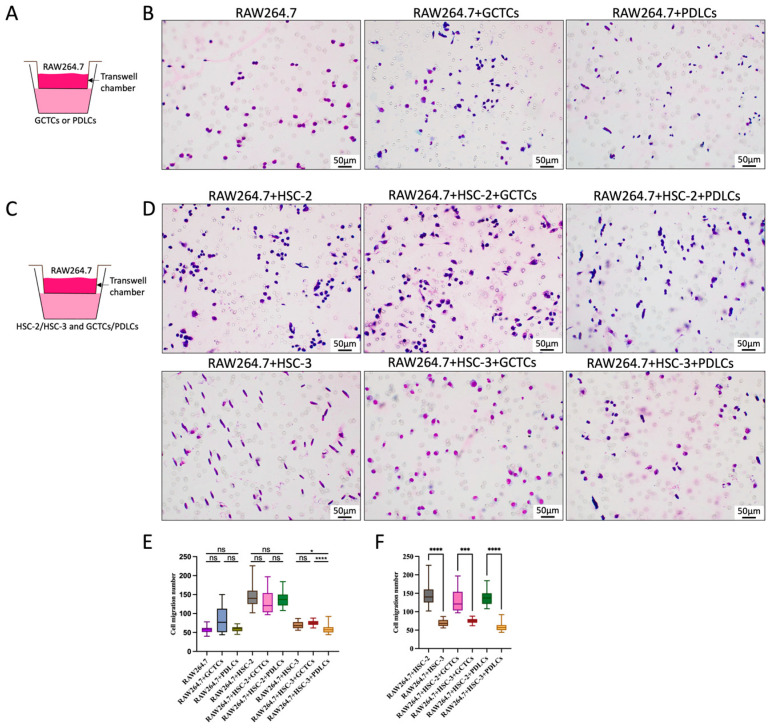
Effects of interaction between GCTCs/PDLCs and HSC-2/HSC-3 on the migration of RAW264.7 in vitro. (**A**,**C**) The Transwell (migration) assay method is illustrated. (**B**) The effect of GCTCs/PDLCs on the migration of RAW264.7 in vitro. (**D**) The effect of the interaction between GCTCs/PDLCs and HSC-2/HSC-3 on the migration of RAW264.7 in vitro. (**E**) Quantification of RAW264.7 migration number in different groups. RAW264.7 + HSC-3 + GCTCs was markedly higher than that in the RAW264.7 + HSC-3 + PDLCs. (**F**) Comparison of the RAW264.7 migration number between HSC-2 and HSC-3. HSC-2 exhibited a significantly higher migratory capacity than HSC-3. ns *p* > 0.05; * *p* < 0.05; *** *p* < 0.001; **** *p* < 0.0001.

GCTCs and PDLCs were digested and collected using Accutase (Invitrogen; Thermo Fisher Scientific, Inc., Carlsbad, CA, USA), and HSC-2, HSC-3, and RAW264.7 were detached and collected using EDTA (Thermo Fisher Scientific, Inc.) when the confluency of these cells reached approximately 90%. In 24-well plates (Corning, Falcon cell culture inserts; BD Biosciences), the upper chamber of 8 µm Transwell filters without Matrigel was mixed with RAW264.7 cells in α-MEM without FBS at a density of 2 × 10^4^ cells/500 µL. In the bottom chamber, GCTCs and PDLCs (15 × 10^4^ cells) were mixed with HSC-2 or HSC-3 (5 × 10^4^ cells) at a ratio of 3:1 in 500 µL of α-MEM that contained 10% FBS, and the schema of the experimental method is shown in Figure 1A,C. After 1 day of incubation at 37 °C, 5% carbon dioxide, and 95% air in a humidified environment, the upper chambers (the membranes closer to the lower chamber) were stained with the Giemsa staining kit (Diff-Quick, Nanjing Jiancheng Bioengineering Institute, Nanjing, China), according to the kit protocol. The specific method involved placing the upper chambers directly into a 24-well plate containing distilled water and various Giemsa staining kits. Each well was treated for two minutes, after which the membrane was cut for slide mounting. Images of the stained cells were captured using a bright-field microscope at 20× magnification (BX51; Olympus Corporation, Tokyo, Japan). Five images were obtained for each sample to determine the cell migration number. Experiments were performed in triplicate in three independent assays, and the data were analyzed using the ImageJ software (V 1.53 K; National Institutes of Health, Bethesda, MD, USA).

### 2.3. Indirect Co-Culture and Immunofluorescence Staining (If)

**Figure 2:**
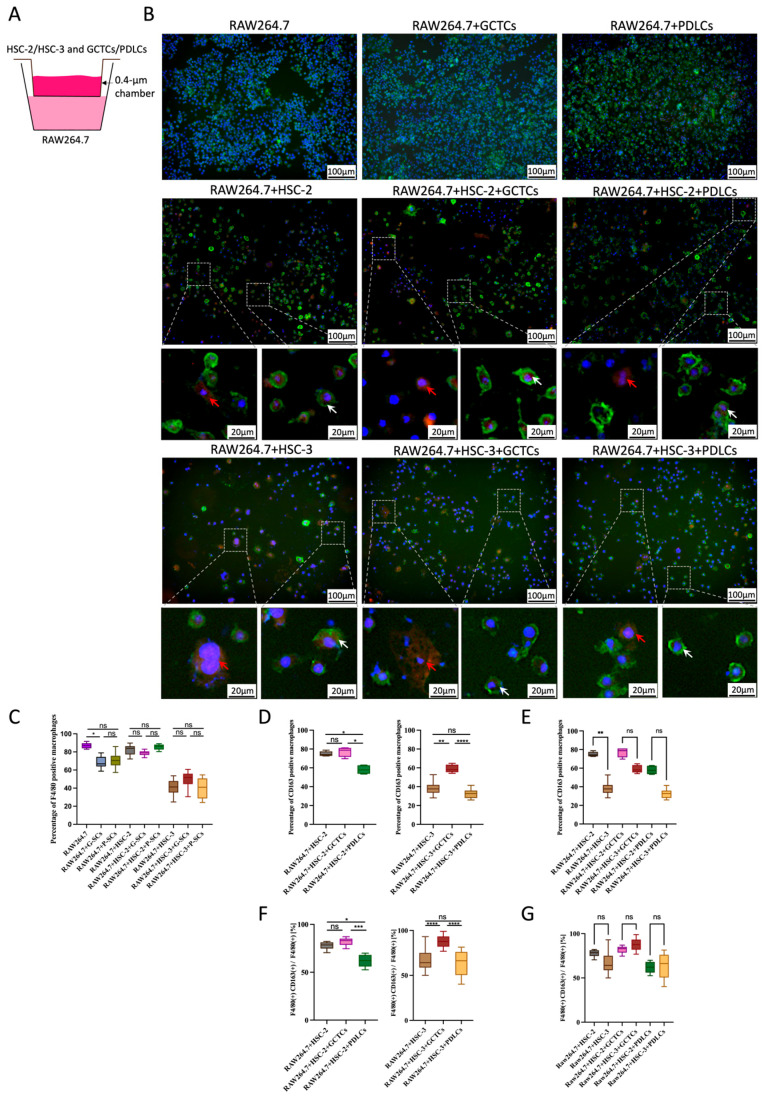
Effects of interaction between GCTCs/PDLCs and HSC-2/HSC-3 on the differentiation of RAW264.7 in vitro. (**A**) The indirect co-culture method is illustrated. (**B**) The effect of GCTCs/PDLCs and the effect of the interaction between GCTCs/PDLCs and HSC-2/HSC-3 on the differentiation of RAW264.7 in vitro. The red arrows indicate CD163(+) macrophages (M2 marker; red), and the white arrows indicate the F4/80(+) CD163(+) macrophages (yellow cells in the merged image). (**C**) Quantification of the percentage of F4/80(+) macrophages in different groups. The percentage of F4/80-positive macrophages showed no significant difference between groups with and without cancer cells. (**D**) Quantification of the percentage of CD163(+) macrophages in different groups. The percentage of CD163(+) macrophages was higher in RAW264.7 + HSC-2 + GCTCs and RAW264.7+HSC-3+GCTCs compared to their respective PDLCs. (**E**) A comparison between HSC-2 and HSC-3. The HSC-2 group showed a higher proportion than the HSC-3 group. (**F**) Quantification of the ratio of F4/80(+) CD163(+) macrophages in F4/80(+) macrophages in different groups. The percentages in RAW264.7 + HSC-2 + GCTCs and RAW264.7 + HSC-3 + GCTCs were markedly higher than those in their respective PDLCs. (**G**) No significant difference in the F4/80(+) CD163(+)/F4/80(+) ratio was observed between the HSC-2 and HSC-3 co-culture groups. ns *p* > 0.05; * *p* < 0.05; ** *p* < 0.01; *** *p* < 0.001; **** *p* < 0.0001.

GCTCs and PDLCs were digested and collected using Accutase (Invitrogen; Thermo Fisher Scientific, Inc.), and HSC-2/HSC-3 and RAW264.7 were digested and collected using EDTA (Thermo Fisher Scientific, Inc.) when the confluency of these cells reached approximately 90%. GCTCs and PDLCs (3.75 × 10^4^ cells) were mixed with HSC-2 or HSC-3 cells (1.25 × 10^4^ cells) at a 3:1 ratio in 500 µL of α-MEM with 10% FBS and were added to a 0.4 µm Transwell filter in a 24-well plate (Greiner Bio-One, Kremsmunster, Austria). The RAW264.7 cells were seeded on the slide glass in the bottom chamber in 500 µL of α-MEM with 10% FBS at a density of 5 × 10^4^ cells/500 µL, and the schema of the experimental method is shown in Figure 2A.

For IF, the slides were washed thrice with TBS (5 min each). The cells were fixed with 4% paraformaldehyde for 10 min and blocked with a blocking solution (DS Pharma Biomedical Co., Ltd., Osaka, Japan) for 20 min. The primary antibodies rat anti-F4/80 (marker of M0 macrophage) (1:10; cat. no. CI:A3-1, Bio-Rad Laboratories, Inc., Hercules, CA, USA), iNOS (marker of M1 macrophage) (1:200; cat. no. 18985-1-AP; Proteintech, Rosemont, IL, USA), and rabbit anti-CD163 (marker of M2 macrophage) (1:200; cat. no. ab182422; Abcam, Cambridge, UK) were added, followed by incubation for 1 h at room temperature. After washing them three times with TBS, the secondary antibodies anti-rat IgG Alexa Fluor 488 (1:200; cat. no. A48262; Thermo Fisher Scientific, Inc.) and anti-rabbit IgG Alexa Fluor 568 (1:200; cat. no. A10042; Thermo Fisher Scientific, Inc.) were added and incubated for 1 h at room temperature in the dark. After washing them with TBS and distilled water thrice, the samples were stained with 0.2 g/mL of DAPI (Dojindo Molecular Technologies, Inc., Kumamoto, Japan). The exposure conditions for green (F4/80), red (iNOS, CD163), and blue (DAPI) were 1.2 s, 0.5 s, and 1/15 s, respectively. The stained cells were photographed using an All-in-One BZ-X700 fluorescence microscope (×40 magnification, Keyence Corporation, Osaka, Japan), as well as setting the positive threshold at 30–124. Independent experiments were repeated in triplicate.

### 2.4. Mouse Model Construction

Animal studies were performed in accordance with the guidelines and regulations of the Animal Care and Use Committee of Okayama University (approval no. OKU-2022354). Anesthesia was administered using isoflurane in accordance with Okayama University Animal Experiment Committee guidelines. Induction was performed at 5% isoflurane, and sedation was maintained at 2–3%. Adequate anesthesia was confirmed by assessing whether the mice could regain a prone position when placed in a supine posture. After the intraperitoneal anesthesia was administered, mixed cells (HSC-2 or HSC-3, 1 × 10^6^ in 100 µL, mixed with GCTCs and PDLCs, 3 × 10^6^ in 100 µL) were injected into the subcutaneous tissue of the heads of 24 healthy female BALB-nu-nu mice (age, 4 weeks; mean body weight, 15 g; Shimizu Laboratory Supplies Co., Ltd., Kyoto, Japan), as previously described [18,19]. After cancer transplantation, the animals were maintained for 4 weeks. If the transplanted tumor reached ≥10 mm in diameter, if the animals lost ≥20% of their body weight within 3 days, or if their food and water intake or exercise capacity decreased, the experiment was terminated immediately and the animal was euthanized. The mice were divided into two experimental groups (n = 4) as follows: the HSC-2-associated experimental group, which included (i) HSC-2 only, (ii) HSC-2+ GCTCs, and (iii) HSC-2 + PDLCs; and the HSC-3-associated experimental group, which included (i) HSC-3, (ii) HSC-3+ GCTCs, and (iii) HSC-3 + PDLCs. The sample size for the animal experiments was determined based on previous studies using similar xenograft models and our own preliminary data, which showed that four mice per group were sufficient to detect statistically significant differences by immunohistochemistry. Although no formal power analysis was conducted, the sample size was deemed appropriate for exploratory purposes and was conducted in accordance with the ethical considerations to minimize animal use.

### 2.5. Hematoxylin and Eosin (HE) Staining

After four weeks, all mice were humanely euthanized by isoflurane overdose (>5%), and cardiac arrest was confirmed via pulse palpation before performing cervical dislocation. Tumor tissues were excised, fixed in 4% paraformaldehyde (PFA; Nacalai Tesque, Kyoto, Japan) for 12 h at room temperature, and then decalcified in 10% ethylenediaminetetraacetic acid (EDTA; Thermo Fisher Scientific, Inc., Waltham, MA, USA) at 4 °C for 4 weeks. The tissue samples were paraffin-embedded, sectioned into 5 μm slices, and stained with HE. Images were obtained using a bright-field microscope (BX51, Olympus Corporation, Tokyo, Japan) at 40× magnification.

### 2.6. Immunohistochemistry (IHC) Staining

Serially prepared 5 μm thick sections were subjected to immunohistochemical staining. CD45 (marker of monocyte), F4/80 (marker of M0 macrophage), iNOS (marler of M1 macrophage), and CD163 (marker of M2 macrophage) antigen repair were conducted in a cooker (Renji Katsuryoku Nabe, Asahi Keikinzoku Kogyo Company, Osaka, Japan) and microwave for 8 or 1 min in 0.01 M tri-sodium citrate buffer (pH 6) and 0.01 M Dako Target Retrieval Solution (pH 9; cat. no. S2367; Agilent Technologies, Inc., Santa Clara, CA, USA). They were then blocked with 10% normal serum for 20 min at room temperature and incubated overnight at 4 °C with the following primary antibodies—rabbit anti-CD45 (1:200; cat. no. ab10558, Abcam), rat anti-F4/80 (1:10; cat. no. MCA497, Abcam), rabbit anti-iNOS (1:200; cat. no. 18985-1-AP, Abcam, Proteintech), and anti-CD163 (1:200; cat. no. ab182422; Abcam, Cambridge, UK). After thorough washing, all sections were incubated with avidin–biotin complexes (PK-6101, rabbit ABC kit; Vector Laboratories, Inc., Newark, CA, USA) for 1 h at room temperature. Sections were developed using a DAB/H_2_O_2_ mixed solution (Histofine DAB substrate; Nichirei Corporation, Tokyo, Japan), and nucleated, dehydrated, and sealed. The soft tissue invasion area (skin side) and bone invasion area (bottom invasion fronts of the tissues) were photographed using a bright-field microscope (BX51, Olympus Corporation). Ten images (×40 magnification) were obtained for each mouse, and CD45, F4/80, iNOS, and CD163 expressions were detected by counting the number of positive cells/images using Image J software (V 1.53 K; National Institutes of Health).

### 2.7. Statistical Analysis

Statistical analyses were performed using GraphPad Prism version 10 (GraphPad Software, Inc., Boston, MA, USA). The cell experiments were independently repeated thrice, and the animal experiments were repeated using four consistent mice per group. For comparisons among multiple groups, the non-parametric Kruskal–Wallis test was used, followed by the Steel–Dwass post hoc test. A value of *p* < 0.05 was considered statistically significant. The significance levels are indicated in the figures as follows: *p* < 0.05 (*), *p* < 0.01 (**), *p* < 0.001 (***), and *p* < 0.0001 (****).

## 3. Results

### 3.1. Effects of GCTCs/PDLCs and HSC-2/HSC-3 Interaction on RAW264.7 Migration

The Transwell (migration) assay method is illustrated in Figure 1A,C. Firstly, we examined the effect of GCTCs or PDLCs on RAW264.7 migration. The migration cell number of RAW264.7 in the RAW264.7 + GCTCs group was slightly higher than that in the RAW264.7-only group and the RAW264.7 + PDLCs group, with no significant differences between them (Figure 1B,E). Next, we assessed the interaction between HSC-2 and GCTCs/PDLCs. In the HSC-2-associated experimental group (RAW264.7 + HSC-2, RAW264.7 + HSC-2 + GCTCs, and RAW264.7 + HSC-2 + PDLCs), there were no significant differences in any group (Figure 1D,E). In contrast, in the HSC-3-associated groups, we observed a more pronounced trend. The migration cell number of RAW264.7 in the RAW264.7 + HSC-3 + GCTCs group was the highest, followed by the RAW264.7 + HSC-3 group, and that of RAW264.7 in the RAW264.7 + HSC-3 + PDLCs group was the lowest (Figure 1D,E). Thus, the interaction between GCTCs and HSC-3 promoted the migration of RAW264.7 in vitro, whereas PDLCs inhibited it. Finally, when comparing the HSC-2- and HSC-3-associated groups overall, the number of migration RAW264.7 cells was significantly higher in the HSC-2-associated experimental group than in the HSC-3-associated experimental group (Figure 1F).

### 3.2. Effects of GCTCs/PDLCs and HSC-2/HSC-3 Interaction on the Differentiation of RAW264.7 into M0 or M2 Macrophages

The indirect co-culture method is shown in Figure 2A. Firstly, the effect of GCTCs and PDLCs on the differentiation of RAW264.7 into M0 macrophages was assayed by the indirect co-culture experiment. Both GCTCs and PDLCs differentiated more than 60% of RAW264.7 into F4/80 (+) macrophages, and there was no significant difference in the effect of differentiation into RAW264.7 between the GCTCs and PDLCs (Figure 2B, upper row, and Figure 2C). The F4/80-positive percentage was not significantly different within the experimental groups in either the HSC-2- or HSC-3-associated experimental groups, and the F4/8-positive percentage was significantly higher in the HSC-2-associated experimental group than in the HSC-3-associated experimental group (Figure 2B, upper row, Figure 2C). Next, the effects of the interaction between the GCTCs/PDLCs and HSC-2 on the differentiation of RAW264.7 into M2 macrophages were examined. The CD163-positive percentage in the HSC-2-associated experimental group was about 80% for RAW264.7 + HSC-2 and RAW264.7 + HSC-2 + GCTCs and was significantly lower in RAW264.7 + HSC-2 + PDLCs at 60% compared to these two groups (Figure 2D). And the effect of the interaction between the GCTCs/PDLCs and HSC-3 on the differentiation of RAW264.7 into M2 macrophages was also examined. In the HSC-3-associated experimental group, the percentage of CD163-positive cells in RAW264.7 was the highest in RAW264.7 + HSC-2 + GCTCs, followed by RAW264.7 + HSC-3 and RAW264.7 + HSC-3 + PDLCs (Figure 2D). Additionally, the percentage of CD163-positive cells in RAW264.7 was higher in the HSC-2-associated experimental group than in the HSC-3-associated experimental group (Figure 2E). To further support these findings, we quantified the overall M2 differentiation rates. Both the RAW264.7 + HSC-2 + GCTCs and RAW264.7 + HSC-3 + GCTCs groups showed M2 differentiation rates of approximately 80%, which were significantly higher than the 60% observed in the RAW264.7 + HSC-2 + PDLCs and RAW264.7 + HSC-3 + PDLCs groups (Figure 2F), with no significant difference between the HSC-2-associated experimental group and the HSC-3-associated experimental group (Figure 2G).

### 3.3. Effects of the Interaction Between GCTCs/PDLCs and HSC-2/HSC-3 on the Differentiation of RAW264.7 to M1 Macrophages

**Figure 3:**
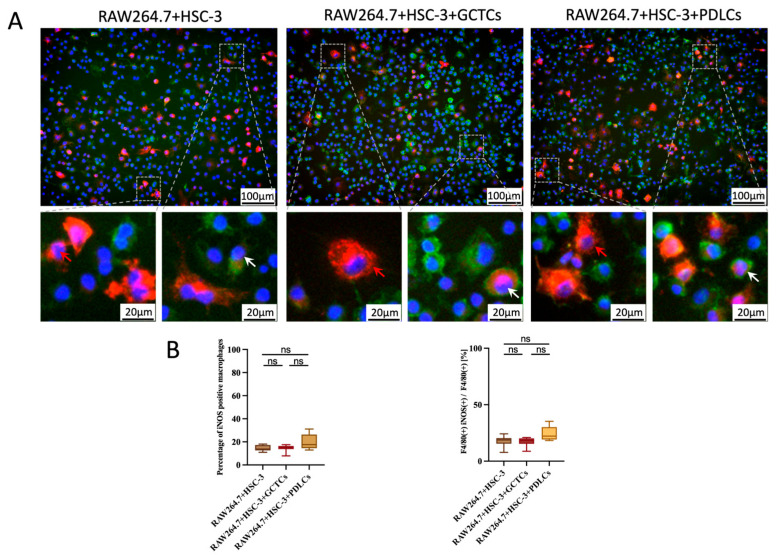
Effects of the interaction between GCTCs/PDLCs and HSC-3 on the differentiation of RAW264.7 in vitro. (**A**) The effect of the interaction between GCTCs/PDLCs and HSC-3 on the differentiation of RAW264.7 in vitro. The red arrows indicate iNOS(+) macrophages (M1 marker; red), and the white arrows indicate the F4/80(+) iNOS(+) macrophages (yellow cells in the merged image). (**B**) Quantification of the percentage of iNOS(+) macrophages and the ratio of F4/80(+) iNOS(+) macrophages in F4/80(+) macrophages in the HSC-3-associated groups. The percentage in the RAW264.7 + HSC-3 + PDLCs group was slightly higher than in the other two groups; however, the difference was not statistically significant. ns *p* > 0.05.

The indirect co-culture method is shown in Figure 2A. The effects of the GCTCs and PDLCs on the differentiation of RAW264.7 in the M1 macrophages were assayed by the indirect co-culture experiment. In the HSC-2-associated experimental group (HSC-2 + RAW264.7, HSC-2 + RAW264.7 + GCTCs, and HSC-2 + RAW264.7 + PDLCs), RAW264.7 did not differentiate into iNOS-positive M1 macrophages. In the HSC-3-associated experimental group, the percentage of iNOS-positive cells in RAW264.7 tended to be higher in the RAW264.7 + HSC-3 + PDLCs group compared to the RAW264.7 + HSC-3 + GCTCs and RAW264.7 + HSC-3 groups. However, the differences were not statistically significant regarding the higher tendency in the RAW264.7 + HSC-3 + PDLCs than the RAW264.7 + HSC-3 + GCTCs and RAW264.7 + HSC-3; however, there was no significant difference (Figure 3A,B).

### 3.4. Effects of the Interaction Between GCTCs/PDLCs and HSC-2/HSC-3 on the Histological Findings in the OSCC TME of the Animal Model

**Figure 4:**
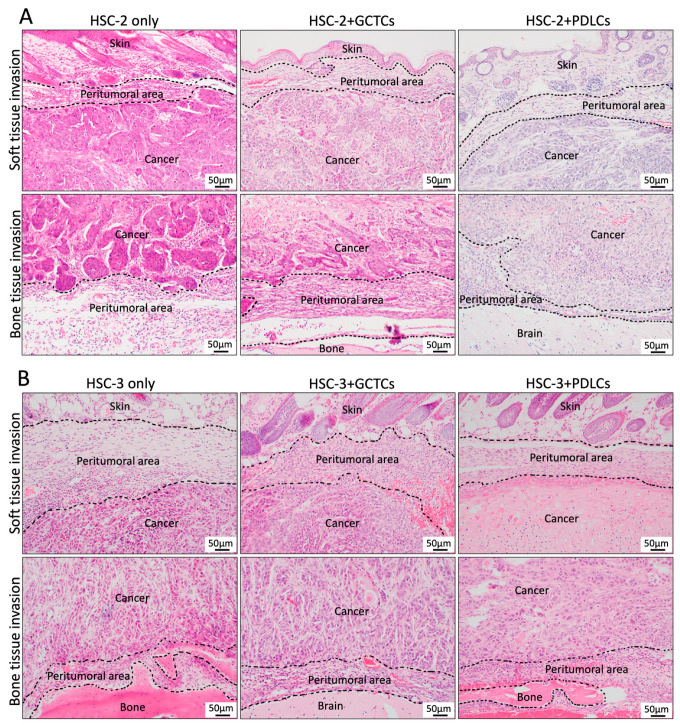
Histological images of the tumor tissue at the soft tissue invasion and bone tissue invasion in the xenograft model. (**A**) Tumor nests were observed at the invasive front, and those in the HSC-2-only group appeared larger than in the HSC-2 + GCTCs and HSC-2 + PDLCs groups. (**B**) Tumor nests were observed at the invasive front. The HSC-3-only and HSC-3 + GCTCs groups exhibited cord-like tumor nests in both soft and bone tissue invasion. The HSC-3 + PDLCs group only showed cord-like tumor nests in the bone tissue invasion, while in the soft tissue invasion, the cancer cells showed keratinization.

Firstly, hematoxylin–eosin staining was performed on the resected specimens. In the HSC-2-only group, large tumor nests were observed in the bone and soft tissue invasion area. In the HSC-2 + GCTCs/PDLCs groups, smaller nests were observed at the infiltrative front of the bone and soft tissue area compared to the HSC-2-only group (Figure 4A). In the HSC-3-associated experimental group, both the HSC-3-only group and the HSC-3 + GCTCs group showed cord-like tumor nests in the bone and soft tissue area. In the HSC-3 + PDLCs group, cord-like tumor nests were observed in the bone tissue area, similar to the HSC-3-only and HSC-3 + GCTCs groups; however, in the soft tissue area, the cancer cells exhibited keratinization and were a well-differentiated-type cancer (Figure 4B). In all experimental groups, round-shaped mononuclear cell infiltration was observed at the tumor invasion front.

### 3.5. Effects of the Interaction Between GCTCs/PDLCs and HSC-2/HSC-3 on the Infiltration of CD45(+) Monocytes in OSCC TME of Animal Model

**Figure 5:**
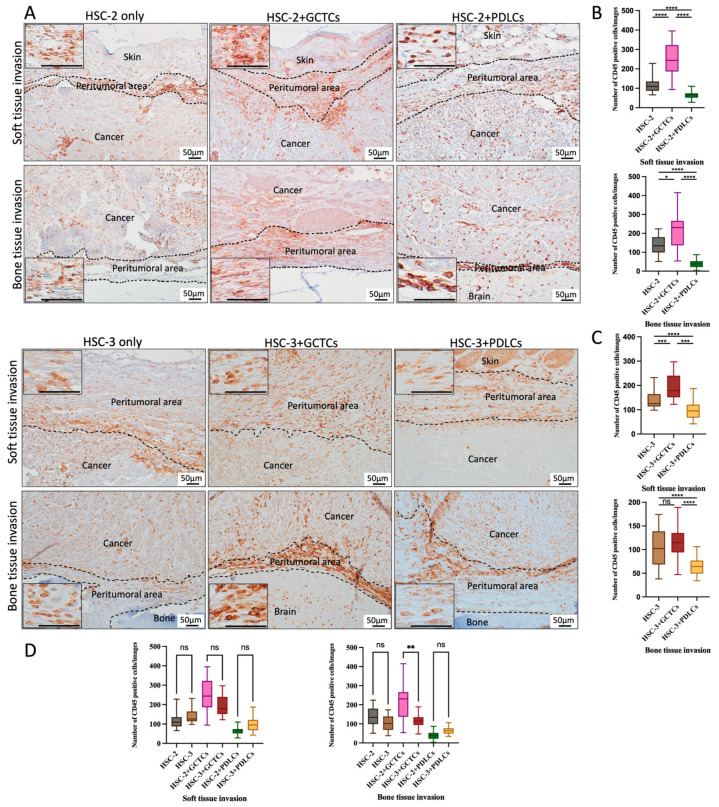
Effects of interaction between GCTCs/PDLCs and HSC-2/HSC-3 on the infiltration of CD45(+) monocytes in the TME of OSCC. (**A**) Immunohistochemical (IHC) staining of CD45(+) monocytes at the invasion front of soft tissue (upper region) and bone tissue (lower region) in xenografted tumors. Insets show the typical morphology of positive monocytes. (**B**,**C**) Quantification of CD45(+) monocytes in the soft (**B**) and bone (**C**) tissue invasion fronts in different groups. HSC-2 + PDLCs and HSC-3 + PDLCs groups were markedly lower than those in the HSC-2 and HSC-3 groups on both the soft tissue and bone tissue sides. In the soft tissue invasion area, both the HSC-2 + GCTCs and HSC-3 + GCTCs groups were higher than those in the HSC-2 and HSC-3 groups. On the bone tissue invasion area, the HSC-2 + GCTCs group was higher than the HSC-2 group, but no significant difference was observed between the HSC-3 + GCTCs and HSC-3 groups. (**D**) A comparison of the number of CD45(+) monocytes between the HSC-2- and HSC-3-associated groups. Notably, the HSC-2 + GCTCs group exhibited a significantly higher number of CD45(+) monocytes than the HSC-3 + GCTCs group. Data are shown as the mean ± SD, n = 4. ns *p* > 0.05; * *p* < 0.05; ** *p* < 0.01; *** *p* < 0.001; **** *p* < 0.0001.

IHC staining was used to test the CD45(+) monocytes so as to assay the effect of the interaction between the GCTCs/PDLCs and HSC-2/HSC-3 cells on the infiltration of CD45(+) monocytes in the soft tissue and bone tissue invasion areas in front of the OSCC (Figure 5A). The infiltration cell number of CD45(+) monocytes in the HSC-2-associated experimental group was the highest in the HSC-2 + GCTCs group, followed by the HSC-2-only and HSC-2 + PDLCs groups in both soft tissue and bone tissue invasion areas, and there was a significant difference in each group (Figure 5B).

In the soft tissue invasion area of the HSC-3-associated experimental group, the infiltration cell number of CD45-positive monocytes at the tumor invasion front showed a similar trend to that of the HSC-2-associated experimental group, the infiltration cell number of CD45-positive monocytes was the highest in the HSC-3 + GCTCs group, which was followed by the HSC-3-only and HSC-3 + PDLCs groups in both the soft tissue and bone tissue invasion areas—and there was a significant difference in each group (Figure 5C). In the bone tissue invasion area, the number of CD45(+) monocytes in the HSC-3-only and HSC-3 + GCTCs groups was higher than in the HSC-3 + PDLCs group, with a significant difference; in contrast, there was no significant difference between the HSC-3-only group and the HSC-3 + GCTCs group (Figure 5C). Comparing the HSC-2- and HSC-3-associated experimental groups, the number of CD45(+) monocytes infiltrating the bone tissue invasion area in the HSC-2 + GCTCs group was significantly higher than that in the HSC-3 + GCTCs group. No significant differences were observed among the other groups (Figure 5D).

In the bone and soft tissue invasion areas of all groups, CD45(+) monocytes exhibited oval or spindle shapes and were considered a heterogeneous cell population (Figure 5A inset). Collectively, the interaction between cancer cell lines and GCTCs promoted the infiltration of CD45(+) monocytes in both the soft tissue and bone tissue invasion fronts of OSCC in vivo, whereas the PDLCs inhibited it. 

### 3.6. Effects of the Interaction Between GCTCs/PDLCs and HSC-2/HSC-3 on the Infiltration of F4/80(+) M0 Macrophages in OSCC TME of Animal Model

**Figure 6:**
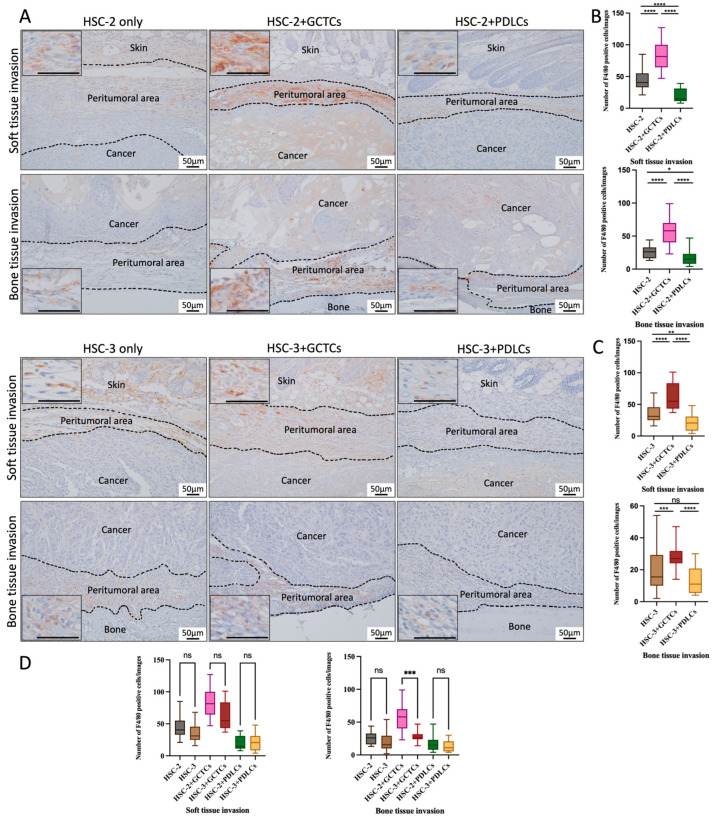
Effects of the interaction between GCTCs/PDLCs and HSC-2/HSC-3 on the infiltration of F4/80(+) M0 macrophages in the TME of OSCC. (**A**) IHC staining for F4/80(+) M0 macrophages at the invasion front of soft tissue (upper region) and bone tissue (lower region) in xenografted tumors. (**B**,**C**) Quantification of F4/80(+) M0 macrophages in the soft (**B**) and bone tissue (**C**) invasion fronts in different groups. The HSC-2 + GCTCs and HSC-3 + GCTCs groups were markedly higher than those in the HSC-2 and HSC-3 groups on both the soft tissue and bone tissue sides. In the soft tissue invasion area, both the HSC-2 + PDLCs and HSC-3 + PDLCs groups were lower than those in the HSC-2 and HSC-3 groups. In the bone tissue invasion area, the HSC-2 + PDLCs group was lower than the HSC-2 group, but no significant difference was observed between the HSC-3 + PDLCs and the HSC-3 groups. (**D**) A comparison of the number of F4/80(+) M0 macrophages in the HSC-2- and HSC-3-associated groups. Most groups showed no significant differences, except for the HSC-2 + GCTCs group, which exhibited a significantly higher number of F4/80(+) M0 macrophages compared to the HSC-3 + GCTCs group. Data are shown as mean ± SD, n = 4. ns *p* > 0.05; * *p* < 0.05; ** *p* < 0.01; *** *p* < 0.001; **** *p* < 0.0001.

IHC staining was used to test the F4/80(+) M0 macrophages so as to assay the effect of the interaction between the GCTCs/PDLCs and HSC-2/HSC-3 cells on the infiltration of F4/80(+) M0 macrophages into the soft and bone invasion fronts of OSCC cells (Figure 6A). The number of F4/80-positive cells at the tumor invasion front was observed in the HSC-2-associated experimental group in the order of the HSC-2 + GCTCs group, HSC-2-only group, and HSC-2 + PDLCs group in both the soft tissue and bone tissue invasion areas, and significant differences were observed between all groups (Figure 6B). In the soft tissue invasion front, the number of F4/80(+) M0 macrophages in the HSC-3 + GCTCs group was higher than that in the HSC-3 group and significantly higher than that in the HSC-3 + PDLCs group (Figure 6C). In the bone tissue invasion front, the number of F4/80(+) M0 macrophages in the HSC-3 + GCTCs group was significantly higher than that in the HSC-3-only and HSC-3 + PDLCs groups, and there was no significant difference between the HSC-3-only and HSC-3 + PDLCs groups (Figure 6C). Comparing the HSC-2- and HSC-3-associated experimental groups, the number of F4/80(+) M0 macrophages infiltrating the bone tissue invasion area in the HSC-2 + GCTCs group was significantly higher than that in the HSC-3 + GCTCs group. No significant differences were observed among the other groups (Figure 6D). Collectively, the interaction between the cancer cell lines and GCTCs promoted the infiltration of F4/80(+) M0 macrophages in both soft and bone tissue invasion fronts of OSCC in vivo, whereas the PDLCs inhibited it.

### 3.7. Effects of the Interaction Between GCTCs/PDLCs and HSC-2/HSC-3 on the Infiltration of CD163(+) M2 Macrophages in OSCC TME of Animal Model

**Figure 7:**
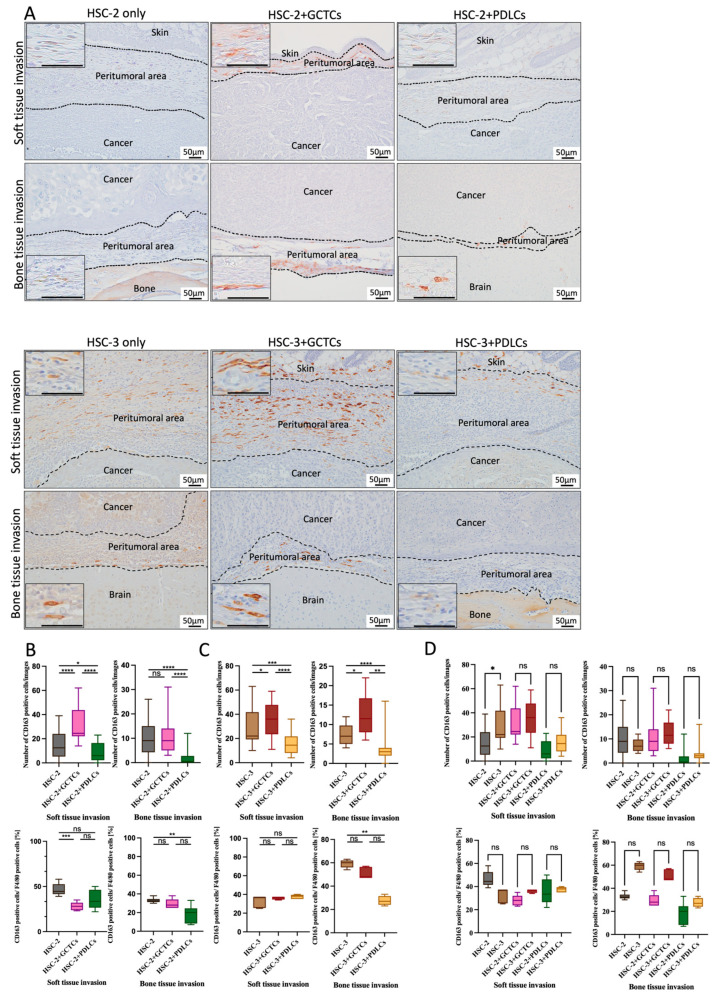
Effects of the interaction between GCTCs/PDLCs and HSC-2/HSC-3 on the infiltration of CD163(+) M2 macrophages in the TME of OSCC. (**A**) IHC staining for CD163(+) M2 macrophages at the invasion front of soft tissue (upper region) and bone tissue (lower region) in xenografted tumors. (**B**,**C**) Quantification of CD163(+) M2 macrophages in the soft and bone tissue invasion fronts in different groups. The upper row represents the number of CD163(+) cells, while the lower row indicates their proportion relative to the F4/80(+) M0 macrophages. In the soft tissue invasion area, the number of CD163(+) cells was highest in the HSC-2 + GCTCs group, followed by the HSC-2 and HSC-2 + PDLCs groups. In the bone tissue invasion area, both the HSC-2 and HSC-2+GCTCs groups showed significantly higher CD163(+) cell numbers than the HSC-2 + PDLC group. As for the proportion of CD163(+) cells among F4/80(+) macrophages, it was lower in the HSC-2 + GCTCs group compared to the HSC-2 group in the soft tissue invasion front, and lowest in the HSC-2 + PDLCs group in the bone tissue invasion front (**B**). The HSC-3 + PDLCs group showed a lower proportion of CD163(+) M2 macrophages among the F4/80(+) macrophages compared to the HSC-3 group in the bone tissue invasion front area. (**D**) A comparison of the number of CD163(+) M2 macrophages in the HSC-2- and HSC-3-associated groups. The upper row represents the number of CD163(+) cells, while the lower row indicates their proportion relative to F4/80(+) M0 macrophages. Overall, no significant differences were observed between the groups, except for a slightly higher number of CD163(+) M2 macrophages in the HSC-3 group compared to the HSC-2 group. Data are shown as the mean ± SD, n = 4. ns *p* > 0.05; * *p* < 0.05; ** *p* < 0.01; *** *p* < 0.001; **** *p* < 0.0001.

IHC staining was used to test the CD163(+) M2 macrophages so as to assay the effect of the interaction between GCTCs/PDLCs and HSC-2/HSC-3 cells on the infiltration of CD163(+) M2 macrophages into the soft and bone tissue invasion fronts of OSCC (Figure 7A). The number of CD163(+) M2 macrophages in the HSC-2 + GCTCs group was the highest in the HSC-2-associated experimental group in the soft tissue invasion area, followed by the HSC-2-only and HSC-3 + PDLCs groups. The number of CD163(+) M2 macrophages in the HSC-2-only group was significantly higher than that in the HSC-2 + PDLCs group. In the bone tissue invasion area, the number of CD163(+) M2 macrophages in the HSC-2-only and HSC-3 + GCTCs groups was significantly higher than that of the HSC-2 + PDLCs group (Figure 7B upper row). The proportion of CD163(+) M2 macrophages among the F4/80(+) M0 macrophages was also analyzed. In the soft tissue invasion areas, the HSC-2 + GCTC group showed a lower proportion compared to the HSC-2-only group. In the bone tissue invasion area, the HSC-2 + PDLCs group exhibited a lower proportion than both the HSC-2-only and HSC-2 + GCTCs groups (Figure 7B, lower row). Collectively, the interaction between HSC-2 and GCTCs promoted the infiltration of CD163(+) M2 macrophages into both soft and bone tissue invasion fronts of OSCC in vivo, whereas the PDLCs inhibited it. Even in the HSC-3-associated experimental group, the same trend was observed as in the HSC-2 experimental group, and this trend was more pronounced in the bone tissue invasion front, especially in the HSC-2 + PDLCs group (Figure 7C upper row). Regarding the proportion of CD163(+) M2 macrophages among F4/80(+) M0 macrophages, the HSC-3 + PDLCs group was lower than the HSC-3 group in the bone tissue invasion area. No significant differences were observed among the other groups (Figure 7C lower row). Comparing the HSC-2- and HSC-3-associated experimental groups, the number of CD163(+) cells infiltrating the HSC-3-associated experimental group tended to be higher than that of the HSC-2-associated experimental group, without a significant difference. A similar trend was observed in the proportion of CD163(+) M2 macrophages among the F4/80(+) M0 macrophages (Figure 7D).

### 3.8. Effects of the Interaction Between GCTCs/PDLCs and HSC-2/HSC-3 on the Infiltration of iNOS(+) M1 Macrophages in OSCC TME of Animal Model

**Figure 8:**
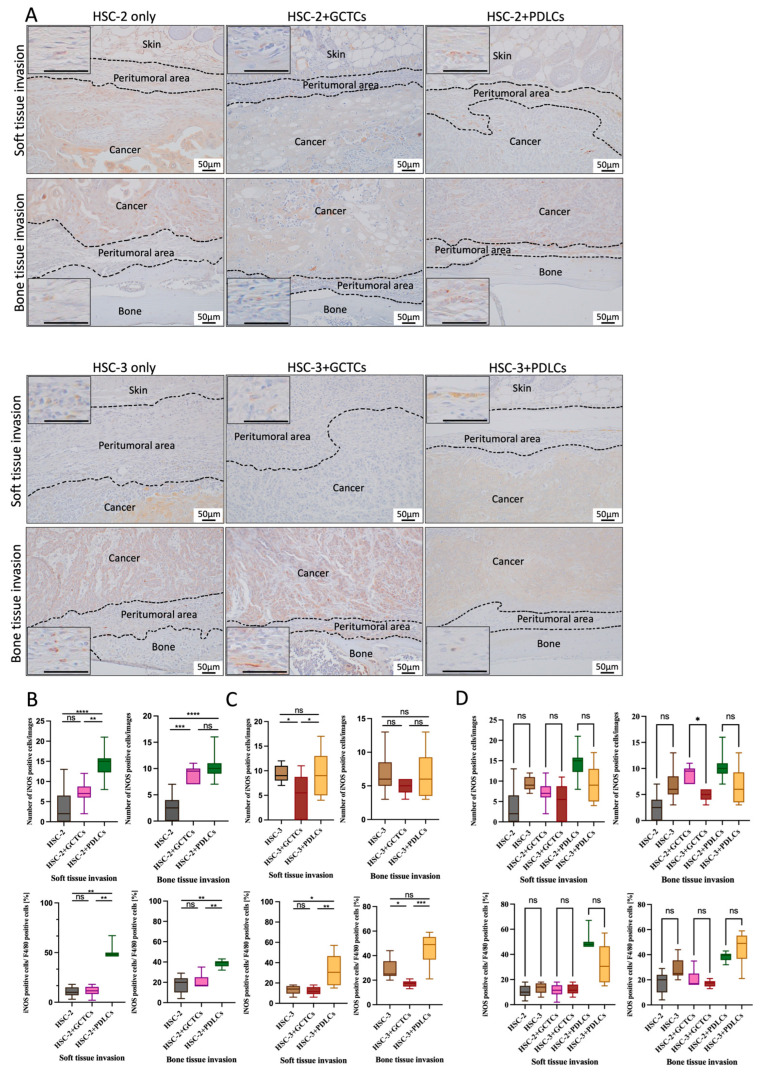
Effects of interaction between GCTCs/PDLCs and HSC-2/HSC-3 on the infiltration of iNOS(+) M1 macrophages in the TME of OSCC. (**A**) IHC staining for iNOS(+) M1 macrophages at the invasion front of soft tissue (upper region) and bone tissue (lower region) in xenografted tumors. (**B**,**C**) Quantification of iNOS(+) M1 macrophages in the soft and bone tissue invasion fronts in different groups. The upper row shows the number of iNOS(+) cells, and the lower row shows their proportion among F4/80(+) M0 macrophages. The HSC-2 + PDLCs group exhibited higher iNOS(+) M1 macrophage numbers than both the HSC-2 and HSC-2 + GCTCs groups in both the soft and bone tissue invasion fronts. A similar trend was observed in the proportion of iNOS(+) M1 macrophages. In the HSC-3-associated experimental group, the iNOS(+) cell numbers in the HSC-3 and HSC-3 + PDLCs groups were higher than in the HSC-3 + GCTCs group. The HSC-3 + PDLCs group also showed a higher proportion of iNOS(+) cells among the F4/80(+) macrophages compared to the other two groups, while in the bone tissue, the HSC-3 + GCTCs group showed a lower proportion than the HSC-3 group. (**D**) A comparison of the iNOS(+) M1 macrophage numbers and proportions between the HSC-2- and HSC-3-associated experimental groups. The upper panel shows the total number of CD163(+) M2 macrophages, while the lower panel illustrates their proportion relative to F4/80(+) M0 macrophages. Overall, no statistically significant differences were observed among the groups. However, the HSC-3 group exhibited a slightly higher number of CD163(+) M2 macrophages compared to the HSC-2 group. Data are shown as mean ± SD, n = 4. ns *p* > 0.05; * *p* < 0.05; ** *p* < 0.01; *** *p* < 0.001; **** *p* < 0.0001.

IHC staining was used to test the iNOS(+) M1 macrophages so as to assay the effect of the interaction between the GCTCs/PDLCs and HSC-2/HSC-3 on the infiltration of iNOS(+) M1 macrophages into the soft and bone tissue invasion fronts of OSCC (Figure 8A). The number of iNOS(+) M1 macrophages in the HSC-2 + PDLCs group was higher than that in the HSC-2-only and HSC-2 + GCTCs groups in the soft and bone tissue invasion fronts (Figure 8B upper row). The proportion of iNOS(+) M1 macrophages among the F4/80(+) M0 macrophages was also analyzed. In both the soft tissue and bone tissue invasion areas, the HSC-2 + PDLC group showed a markedly higher proportion than both the HSC-2 and HSC-2 + GCTC groups (Figure 8B, lower row).The number of iNOS(+) M1 macrophages in the HSC-3-only and HSC-3 + PDLCs groups was higher than that in the HSC-3 + GCTCs group in the soft and bone tissue invasion fronts (Figure 8C, upper row). Regarding the proportion of iNOS(+) M1 macrophages among the F4/80(+) M0 macrophages, the HSC-3 + PDLC group showed a markedly higher proportion than both the HSC-3 and HSC-3 + GCTC groups. In the bone tissue invasion area, the HSC-3 + GCTCs group was lower than the HSC-3 group (Figure 8C, lower row). In both the soft and bone tissue invasion areas, the cancer cells alone tended to have a higher number of iNOS-positive M1 macrophages in the HSC-3-associated experimental group than in the HSC-2-associated experimental group; however, there was no significant difference in either experimental group. On the other hand, in the GCTCs and PDLCs added groups, the HSC-2-associated experimental group tended to have a higher number of iNOS-positive M1 macrophages compared to the HSC-3-associated experimental group, and a significant difference was observed in the GCTCs experimental group in the bone tissue invasion area. Although the proportion was higher in the PDLC-added groups compared to both the cancer-only groups and GCTC-added groups, there was no significant difference (Figure 8D).

## 4. Discussion

In this study, the fibrous cells established from gingival connective tissue interacting with cancer cell lines accumulated monocyte macrophages in the TME and induced the differentiation of monocyte macrophages into M2 macrophages. In contrast, the fibrous cells established from the periodontal ligament interacting with cancer cell lines inhibited the accumulation of monocyte macrophages in the TME and suppressed the differentiation of monocyte macrophages into M2 macrophages, and concurrently induced a relative increase in M1 macrophages, suggesting a shift in the polarization balance toward an antitumor phenotype. This is the first study to investigate the interaction between cancer parenchyma and peritumoral tissue on the accumulation and differentiation of TAMs, and it is also the first study to demonstrate that the nature of the peritumoral tissue directly affects the accumulation and differentiation of TAMs. This study differs significantly from previous studies on TAMs, which have focused exclusively on cancer parenchyma.

The interaction between GCTCs and HSC-2/HSC-3 promoted the infiltration of CD45(+) monocytes/F4/80(+) M0 macrophages into the OSCC TME, whereas the interaction between PDLCs and HSC-2/HSC-3 inhibited it both in vitro and in vivo. Macrophages are a major population of infiltrating leukocytes that are associated with solid tumors [20]. TAMs are replenished in the TME from surrounding tissues by the cancer itself through the secretion of chemotactic molecules, and monocytes circulating in the bloodstream infiltrate the TME and mature into TAMs [21]. A study using a mouse model suggested that the tumor-infiltrating monocyte pool is predominantly Ly-6C + CX3CRlow and that TAM monocyte precursors are mostly Ly-6Chigh cells that were F4/80 positive [22]. Therefore, the macrophages originate from the monocytes in the circulating blood, which plays a significant role in cancer progression [14,23,24,25]. This infiltration of monocytes is greatly influenced by the interaction between the parenchymal tissue and tumor cells. Monocyte infiltration is closely associated with microvascular density (MVD) in various cancers, such as pancreatic and bladder cancers [26,27]. A previous study revealed a difference in the ability to induce MVD based on the characteristics of the cancer stroma [28]; therefore, the nature of the tumor stroma could be related to the aggregation of monocytes from which the TAM is derived. The results revealed that the interaction between GCTCs and HSC-2/HSC-3 promoted the infiltration of F4/80(+) cells, which differentiated into TAMs or myeloid-derived suppressor cells [29,30], both of which have been reported to have a poor prognosis for OSCC [31].

Co-culture of GCTCs and HSC-2/HSC-3 promotes the differentiation of monocytes into M2 TAMs. TAMs are characterized by remarkable plasticity and can acquire various intermediate activation stages by simultaneously expressing markers that are associated with both the M1 and M2 phenotypes, although their terminal polarization into M1 or M2 remains a key determinant of their functional roles [32]. In many tumors, the number of M2-type TAM clusters acutely reflects the degree of malignancy [33,34,35]. In most solid tumors, the macrophage balance in OSCC tends toward the M2 phenotype [36,37,38,39,40]. Co-culture of a monocyte/macrophage-like cell line (RAW264.7) with a conditioned medium that was derived from the OSCC cell line promoted the expression of tumor-promoting cytokines and chemokines that are associated with the M2 phenotype [41]. These results are consistent with those of our in vitro co-culture experiments. In our previous study, the interaction of cancer cells with GCTCs decreased the degree of differentiation (changing the poorly differentiated type of cancer), and the interaction of cancer cells with PDLCs changed cancer cells to a well-differentiated type of cancer [16]. In general, the infiltration number of TAMs into cancer tissues is low in well-differentiated OSCC, whereas the infiltration number of TAMs into cancer tissues is marked in poorly differentiated OSCC [42]. Therefore, the results of the present study are consistent with those of our previous studies and those of other studies on OSCC. However, co-cultures of PDLCs and HSC-2/HSC-3 induced less differentiation toward M2-type TAMs compared to the HSC-2/HSC-3-alone group. In addition, a relative increase in iNOS(+) M1 macrophages was observed in these co-culture conditions, suggesting that PDLCs may influence TAM polarization by not only suppressing the tumor-promoting M2 phenotype but also by enhancing M1-type activation under certain microenvironmental conditions. Stem cell sensation in the periodontal ligament shifts macrophage polarization toward M2 and is involved in tissue repair. Periodontal ligament stem cells (PDLSCs) were able to induce M2 macrophage polarization instead of M1 polarization, and they were also capable of enhancing M2 macrophage polarization induced by IL-4 and IL-13. The JNK pathway was involved in promoting M2 macrophage polarization. PDLSC condition medium (PDLSC-CM) enhances periodontal regeneration by suppressing the inflammatory response through TNF-α production, and transplantation of PDLSC-CM could be a novel approach for periodontal regenerative therapy [43,44,45]. Conversely, the periodontal ligament expresses M1 macrophage inducers such as iNOS when subjected to mechanical stresses, including occlusal or orthodontic forces [46]. Thus, it has been suggested that the periodontal ligament polarizes monocyte macrophages toward M1 rather than M2 when they compete with cancer cells, which is consistent with our data.

Notably, we observed distinct functional differences between HSC-2 and HSC-3 cells in both the in vitro and in vivo experiments. In the Transwell migration assays, HSC-2 cells consistently attracted more RAW264.7 cells compared to the HSC-3 cells, not only in the single-culture condition but also when co-cultured with either GCTCs or PDLCs. This suggests that HSC-2 cells possess a stronger chemotactic ability toward monocytes/macrophages. In terms of polarization, HSC-2 induced a higher M2 conversion rate than HSC-3, although no significant differences were observed in the proportion of F4/80(+) macrophages between the groups. In the animal model, tumors derived from the HSC-2 + GCTC group exhibited a higher infiltration of CD45(+) monocytes and F4/80(+) macrophages than those in the HSC-3 + GCTC group, which is consistent with the in vitro migration results. Furthermore, the number of iNOS(+) M1 macrophages was significantly higher in the HSC-2 + GCTC group, whereas the M2 macrophage numbers did not differ markedly between the co-culture groups, aside from a slight increase in the HSC-3 group. The overall M1/M2 conversion rates remained comparable across groups. Taken together, these findings suggest that HSC-2, despite being less aggressive than HSC-3 in terms of histological grade, may more potently influence the tumor immune microenvironment by enhancing monocyte recruitment and supporting M1 macrophage polarization. These differences could be due to cell line-specific secretion profiles of chemokines or other immunomodulatory factors and highlight the importance of considering tumor heterogeneity when evaluating TAM behavior and therapeutic strategies [47].

In this study, we investigated the infiltration of TAMs into two areas: one on the skin side and the other on the bone tissue side. In the soft tissue invasion front, significant differences were observed between the GCTCs and PDLCs groups. However, no significant differences were observed between these groups at the bone tissue invasion front, which may be owing to complex structures such as bone, muscle, and brain in the bone tissue invasion front. In our previous study, we reported that cancer stroma promotes bone destruction by inducing osteoclasts on the bone surface [17]. In addition, interactions between the HSC-2/HSC-3 cells and normal stroma promoted the fusion of macrophages into multinucleated giant cells. Therefore, it was suggested that factors released from the bone destroyed by the cancer/stroma complex caused the assembled macrophages to differentiate into osteoclasts or promoted the fusion of macrophages into multinucleated giant cells rather than into M2 macrophages.

However, it is important to note that our study had some limitations. TAMs represent a variety of macrophage subsets that change in response to the cytokine balance within the TME, primarily with an M2-like phenotype [48]. TAMs also exhibit both M1 and M2 polarization features [49,50]. Qian et al. proposed the following six TAM phenotypes: activated, immunosuppressive, angiogenic, metastasis-associated, perivascular, and invasive macrophages [51]. Additionally, Komohara et al. have suggested that TAMs contain various macrophage phenotypes with a wide range of polarization statuses stimulated by multiple signaling pathways in the TME [52]. In our current study, TAMs were identified by single-marker immunohistochemistry, which allowed us to evaluate the distribution of M1- and M2-type macrophages in different experimental groups. However, this method precludes the assessment of marker co-expression within the same cell, which is a limitation. Future studies using multiplex or colocalization-based immunofluorescence techniques could provide more detailed insight into TAM heterogeneity and the coexistence of polarization states in situ. In addition, while the current study focused on the effects of perioral stromal cells on macrophages, the direct impact of perioral stromal cells on OSCC cells themselves, such as the changes in cytokine secretion or proliferative capacity, was not addressed. Our previous study demonstrated that stromal origin could influence OSCC cell differentiation and malignant behavior; however, further investigation is needed to determine whether these stromal components also modulate tumor-derived cytokine profiles or proliferative signaling pathways [16]. First, the factors that regulate the cytokine balance involved in TAM polarization when the cancer stroma and parenchyma interact must be determined. Second, additional research is required to elucidate the infiltrative TAM subsets by single-cell RNA sequencing and cDNA arrays because TAM-specific markers have not been identified.

## 5. Conclusions

The interaction between GCTCs and HSC-2/HSC-3 promoted the infiltration of TAMs into the TME of OSCC, whereas PDLCs inhibited it. The interaction between GCTCs and HSC-2/HSC-3 had a stronger effect on the differentiation of TAMs than that between PDLCs. These findings reveal the role and function of the peritumoral issue in OSCC progression, providing a new regulatory mechanism for OSCC development.

## Data Availability

The raw data supporting the conclusions of this article will be made available by the authors upon request.
